# IscR Regulation of Capsular Polysaccharide Biosynthesis and Iron-Acquisition Systems in *Klebsiella pneumoniae* CG43

**DOI:** 10.1371/journal.pone.0107812

**Published:** 2014-09-19

**Authors:** Chien-Chen Wu, Chien-Kuo Wang, Yu-Ching Chen, Tien-Huang Lin, Tzyy-Rong Jinn, Ching-Ting Lin

**Affiliations:** 1 Institute of Biochemistry and Molecular Biology, National Yang-Ming University, Taipei, Taiwan, Republic of China; 2 Department of Biotechnology, Asia University, Taichung, Taiwan, Republic of China; 3 Department of Biomedical Informatics, Asia University, Taichung, Taiwan, Republic of China; 4 Division of Urology, Taichung Tzu Chi Hospital, The Buddhist Tzu Chi Medical Foundation, Taichung, Taiwan, Republic of China; 5 Graduate Institute of Chinese Medicine, School of Chinese Medicine, China Medical University, Taichung, Taiwan, Republic of China; Queen's University Belfast, United Kingdom

## Abstract

IscR, an Fe–S cluster-containing transcriptional factor, regulates genes involved in various cellular processes. In response to environmental stimuli such as oxidative stress and iron levels, IscR switches between its holo and apo forms to regulate various targets. IscR binding sequences are classified into two types: the type 1 IscR box that is specific for holo-IscR binding, and the type 2 IscR box that binds holo- and apo-IscR. Studying *Klebsiella pneumoniae* CG43S3, we have previously shown that iron availability regulates capsular polysaccharide (CPS) biosynthesis and iron-acquisition systems. The present study investigated whether IscR is involved in this regulation. Compared with that in CG43S3, the amount of CPS was decreased in AP001 (Δ*iscR*) or AP002 (*iscR*_3CA_), a CG43S3-derived strain expressing mutated IscR mimicked apo-IscR, suggesting that only holo-IscR activates CPS biosynthesis. Furthermore, a promoter-reporter assay verified that the transcription of *cps* genes was reduced in AP001 and AP002. Purified IscR::His_6_, but not IscR_3CA_::His_6_, was also found to bind the predicted type 1 IscR box specifically in the *cps* promoter. Furthermore, reduced siderophore production was observed in AP004 (Δ*fur*-Δ*iscR*) but not in AP005 (Δ*fur*-*iscR*_3CA_), implying that apo-IscR activates iron acquisition. Compared with those in AP004, mRNA levels of three putative iron acquisition systems (*fhu*, *iuc*, and *sit*) were increased in AP005, and both purified IscR::His_6_ and IscR_3CA_::His_6_ bound the predicted type 2 IscR box in the *fhuA*, *iucA*, and *sitA* promoters, whereas IscR_3CA_::His_6_ displayed a lower affinity. Finally, we analyzed the effect of external iron levels on *iscR* expression. The transcription of *iscR* was increased under iron-depleted conditions as well as in AP001 and AP002, suggesting an auto-repression exerted by apo-IscR. Our results show that in *K. pneumoniae*, IscR plays a dual role in the regulation of CPS biosynthesis and iron-acquisition systems in response to environmental iron availability.

## Introduction

*Klebsiella pneumoniae* is a rod-shaped, gram-negative bacterium that causes community-acquired diseases including pneumonia, bacteremia, septicemia, and urinary and respiratory tract infections that occurr particularly in immune-compromised patients [1]. In Asian countries, especially in Taiwan and Korea, *K. pneumoniae* is the predominant pathogen responsible for pyogenic liver abscesses in diabetic patients [2], [3], [4]. Among the virulence factors identified in *K. pneumoniae*, capsular polysaccharide (CPS) is considered the major determinant for *K. pneumoniae* infections. Pyogenic liver abscess isolates often carry heavy CPS loads that protect bacteria from phagocytosis and from being killed by serum factors [5], [6]. Apart from its anti-phagocytic function, *Klebsiella* CPS also promotes bacterial colonization and biofilm formation at infection sites [7], [8], [9].

Our previous studies have demonstrated that CPS biosynthesis in *K. pneumoniae* is repressed in iron-replete conditions, and this regulation is controlled by an iron uptake regulator (Fur) [10]. Under iron-replete conditions, dimeric Fur in complex with Fe(II) indirectly activates *K. pneumoniae* CPS biosynthesis through transcriptional factors RmpA and RcsA and a small non-coding RNA, RyhB [10], [11]. The transcription of *cps* genes is directly regulated by RmpA and RcsA but appears to be indirectly regulated by RyhB. These findings indicate that environmental iron availability influences *K. pneumoniae* CPS biosynthesis through multiple regulators.

To maintain iron homeostasis, Fur acts as a master regulator to control iron transport, storage, and metabolism in many gram-negative bacteria including *K. pneumoniae* [11]–[13]. We have previously reported that Fur directly represses at least six of the eight iron acquisition systems in *K. pneumoniae* CG43S3 [10]. In addition to Fur, the transcriptional regulator IscR plays a crucial role in iron metabolism. IscR regulates the biosynthesis of Fe-S clusters, which are key cofactors of proteins intervening in various cellular processes in bacteria [12], [13]. Fe-S clusters can be generally classified into two types, rhombic [2Fe-2S] and cubic [4Fe-4S], which have either ferrous (Fe^2+^) or ferric (Fe^3+^) iron and sulphide (S^2−^) [14], [15]. IscR is itself a [2Fe-2S] cluster-containing protein encoded by the first gene of the *iscRSUA* operon. The switch between the [2Fe-2S] holo and apo forms of IscR is believed to be influenced by environmental conditions such as oxidative and nitric oxide stress and cellular iron levels [13], [16], [17], [18]. Moreover, holo- and apo-IscR have been shown to regulate different target genes, suggesting that the presence of the [2Fe-2S] cluster affects the regulatory specificity of IscR [18], [19], [20], [21].

Transcriptomic analysis has identified 40 genes in 20 predicted operons, which are regulated by IscR under aerobic and anaerobic conditions in *Escherichia coli* [19]. This analysis has also revealed two classes of IscR binding sites (IscR boxes). Type 1 IscR box consists of a 25-bp sequence interacted with holo-IscR, whereas type 2 IscR box consists of a 26-bp sequence interacted with apo-IscR [19]. Furthermore, a detailed analysis of the type 2 IscR box has verified an IscR binding motif for both holo and apo-IscR binding [21].

In this study, we investigated whether IscR participates in the regulation of CPS biosynthesis and the expression of iron acquisition systems in *K. pneumoniae*. We also analysed the expression of *iscR* in response to various iron levels.

## Results

### IscR activates *K. pneumoniae* CPS biosynthesis in an Fe-S cluster-dependent manner

**Figure 1:**
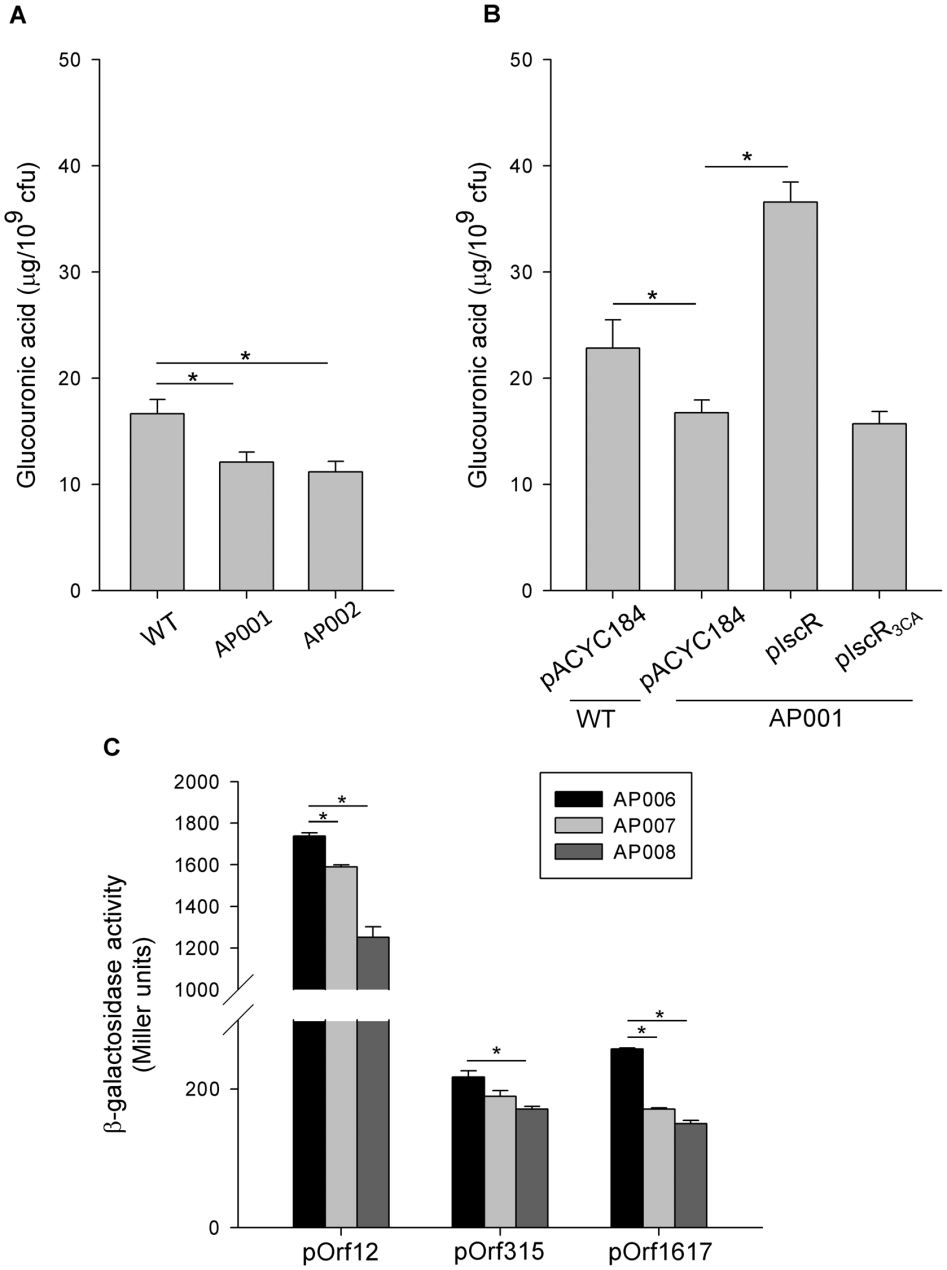
Holo-IscR positively regulates the biosynthesis of CPS. (A) CPS levels of the WT, AP001, and AP002 strains grown in LB broth. (B) CPS levels in WT carrying pACYC184 and AP001 carrying pACYC184, pIscR, or pIscR_3CA_ were determined in LB. Bacterial glucuronic acid content was determined after 16 h of growth. (C) β-Galactosidase activities of *K. pneumoniae* AP006 and isogenic strains (AP007 and AP008) carrying the reporter plasmid pOrf12 (P*_orf1-2_*::*lacZ)*, pOrf315 (P*_orf3-15_*::*lacZ*), or pOrf1617 (P*_orf16-17_*::*lacZ*) were determined using log-phase cultures grown in LB medium. Error bars indicate standard deviations. *P<0.01 compared with the indicated groups.

To study whether IscR regulates *K. pneumoniae* CPS biosynthesis, we determined the amounts of K2 CPS in CG43S3 (wild type [WT]) and AP001 (Δ*iscR*) strains. Compared with the WT, AP001 produced significantly lower amounts of CPS (Fig. 1A), suggesting that IscR activates the biosynthesis of CPS. In *K. pneumoniae*, IscR contains three highly conserved cysteine residues (C92, C98, and C104 in *E. coli* IscR) which are thought to coordinate the [2Fe-2S] cluster [20].

To investigate the role of the [2Fe-2S] cluster in IscR regulation of CPS biosynthesis, we created an *iscR* mutant AP002 (*iscR*_3CA_) by replacing the three cysteines with alanines and tested whether this mutant, which is predicted to encode an IscR lacking an Fe-S cluster, affected CPS biosynthesis. As shown in Fig. 1A, we found that the amount of CPS decreased in the AP002 strain compared with that in the WT, indicating that the regulation of IscR required the [2Fe-2S] cluster. Moreover, *iscR* and *iscR*_3CA_ were respectively cloned into pACYC184, to yield pIscR and pIscR_3CA_, for complementation analysis. Compared with AP001 [pACYC184], AP001 [pIscR] produced a significantly higher amount of CPS, whereas the introduction of pIscR_3CA_ into the AP001 strain did not change the CPS amount (Fig. 1B). These results confirmed that IscR has a positive role in the regulation of CPS biosynthesis and that the presence of the [2Fe-2S] cluster of IscR is essential for this regulation. On the other hand, the CPS amount appeared to obviously increased in AP001 [pIscR], compared with that in WT [pACYC184] (Fig. 1B), which may result from multicopy plasmids are used for complementation. Therefore, we also used single copy constructs to complement the *iscR*-deletion (Methods S1), and the result showed that the expression of *iscR*, but not *iscR*_3CA_, could restore the CPS biosynthesis (Fig. S1).

The K2 *cps* gene cluster of *K. pneumoniae* contains 19 open reading frames (ORFs) organised into 3 transcription units–namely, *orf1–2*, *orf3–15*, and *orf16–17* [22]. To determine the role of IscR in regulating *cps* transcription, we used the reporter plasmids pOrf12 (P*_orf1-2_*::*lacZ*), pOrf315 (P*_orf3-15_*::*lacZ*), and pOrf1617 (P*_orf16-17_*::*lacZ*), each carrying a promoterless *lacZ* gene transcriptionally fused to the putative promoter region of the K2 *cps* gene cluster [23], to transform the *K. pneumoniae* strains AP006 (Δ*lacZ*), AP007 (Δ*lacZ-*Δ*iscR*), and AP008 (Δ*lacZ-iscR*_3CA_). The measurements shown in Fig. 1C reveal that the promoter activity of *orf1–2* and *orf16–17* in AP007 and AP008 was lower than that in AP006 (*P*<0.01). Additionally, the promoter activity of *orf3–15* was apparently decreased in AP008 but slightly decreased in AP007 compared with that in AP006. These results indicated that IscR activates the transcription of *cps* genes in an Fe-S cluster-dependent manner.

### IscR directly binds the promoter of *galF*

**Figure 2:**
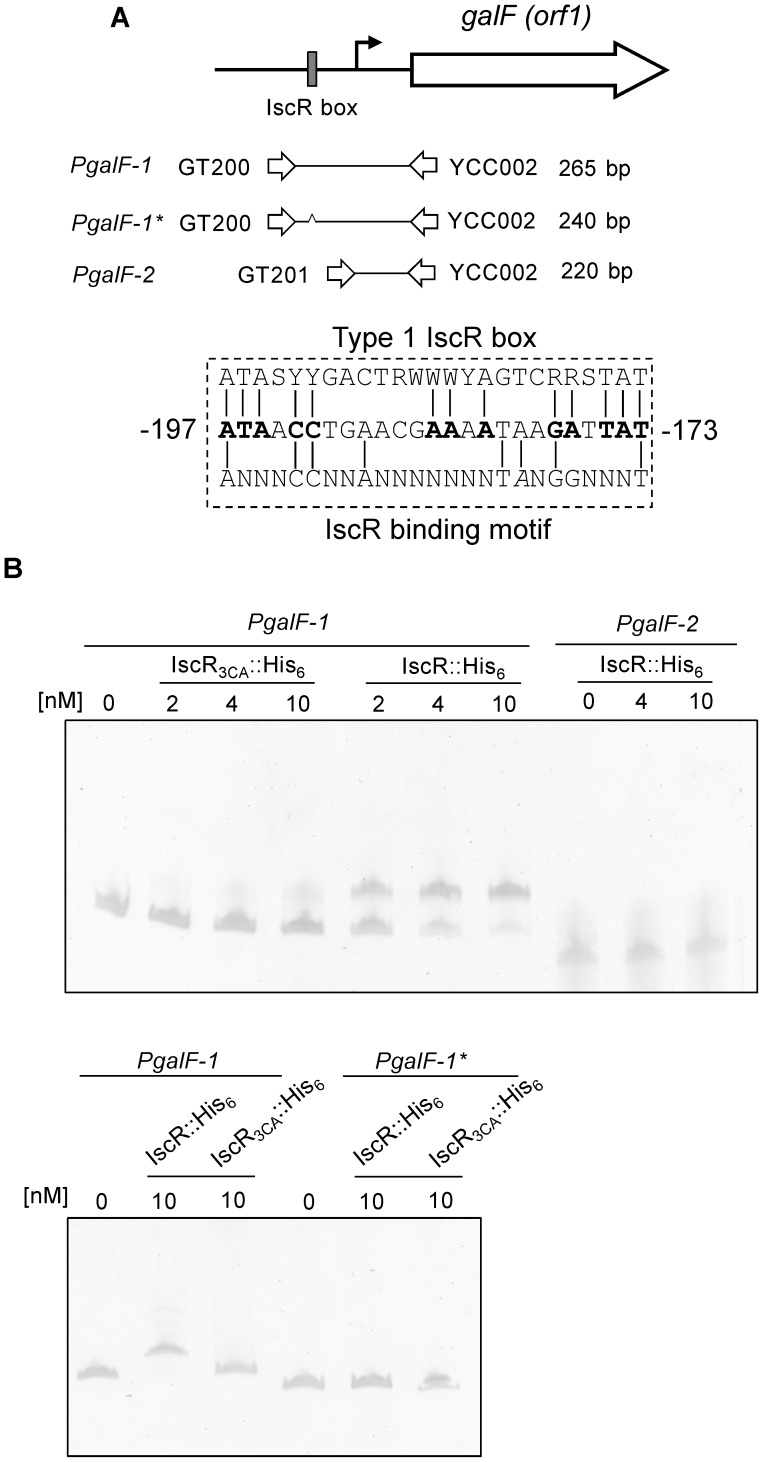
IscR binds directly to P*_galF_*. (A) Diagrammatic representation of the *galF* loci. The primer sets used in PCR amplification of the DNA probes are indicated, and the numbers denote the DNA amplified length. DNA probes are listed on the left. The box in grey indicates the predicted type 1 IscR box. The dashed box indicates the DNA sequence alignment among the predicted type 1 IscR box, the IscR binding motif, and the putative IscR binding sequence in P*_galF_*, and the numbers denote the positions relative to the translational start site. Deletion of the predicted IscR box on PgalF-1* is indicated by a caret. (B) EMSA of IscR recombinant proteins and various DNA fragments of the upstream regions of *galF*. Different concentrations of purified IscR::His_6_ or IscR_3CA_::His_6_ were incubated with 5 ng of DNA fragments, as indicated in the margin. After incubation at room temperature for 30 min, the mixtures were resolved on a 5% non-denaturing polyacrylamide gel. The gel was stained with SYBR Green I dye and photographed.

For further investigation of the mechanism of IscR regulation on *cps* transcription, the sequence of the putative IscR binding site was manually analysed in the three promoter regions of the K2 *cps* gene cluster. As shown in Fig. 2A, we found a putative type 1 IscR box with 52% (13/25 bp) homology to the consensus sequence located between −173 bp and −197 bp relative to the translational start codon of *galF* (*orf1* in the K2 *cps* gene cluster). In addition, the putative IscR binding sequence in P*_galF_* was highly homologous to the IscR-binding motif (5′-AxxxCCxxAxxxxxxxTAxGGxxxT-3′) reported by Nesbit *et al*. [21]. However, no typical IscR binding site was found in the upstream sequence of *wzi* (*orf3* in the K2 *cps* gene cluster) or *manC* (*orf16* in the K2 *cps* gene cluster), suggesting that IscR indirectly regulates the promoter activities of *orf3*-*15* and *orf16*-*17*, which remains to be studied. On the other hand, we hypothesised that IscR binds directly to the promoter region of *galF* to activate gene transcription, and we confirmed this by performing an electrophoretic mobility shift assay (EMSA). As shown in the upper panel of Fig. 2B, purified recombinant IscR::His_6_ protein was able to bind *PgalF-1* but not *PgalF-2*, in which the region containing the putative IscR binding site was deleted. In addition, compared with that of IscR::His_6_, the recombinant [2Fe-2S] clusterless IscR_3CA_::His_6_ had reduced *PgalF-1* binding activity. Furthermore, no obvious interaction between the recombinant IscR proteins and PgalF-1*, the *galF* promoter lacking only the 25-bp predicted IscR box, was found (the lower panel of Fig. 2B). Besides, *PgalF-1* and PgalF-1* DNA showed a slightly different mobility in the gel. These results suggested a direct interaction between IscR and the *galF* promoter and that the [2Fe-2S] cluster of IscR plays a crucial role in this interaction. On the contrary, we also analysed whether recombinant IscR::His_6_ could bind the promoter regions of *wzi* and *manC*. As expected, EMSA showed no obvious DNA-protein complex (data not shown).

### Effect of IscR on normal human serum resistance

**Figure 3:**
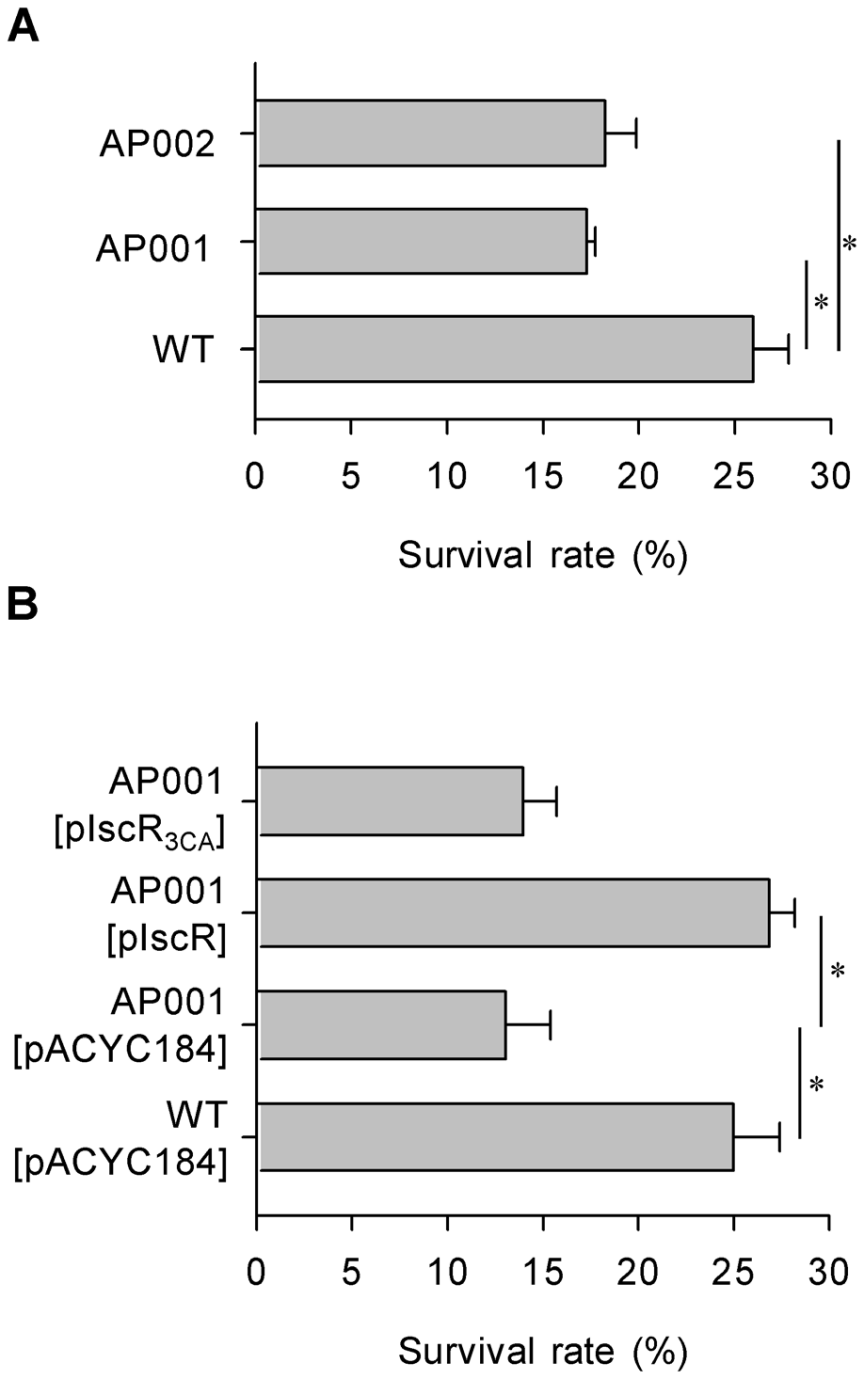
Deletion effect of *iscR* on *K. pneumoniae* susceptibility to normal human serum. The susceptibility to normal human serum of each bacterial mutant (A) and the complement strains (B) indicated in the margin was determined. Bacterial serum resistance was determined using log-phase cultures grown in LB medium. *P<0.01 compared with the indicated groups.

Because CPS acts as a protectant for *K. pneumoniae* against serum factors, we hypothesize that through modulation of CPS levels, IscR affects the ability of *K. pneumoniae* to resist the bactericidal effects of serum. To test this hypothesis, we treated *K. pneumoniae* strains with 75% normal human serum and determined their survival rates. Compared with the WT, the AP001 and AP002 strains had a slightly reduced survival rate (Fig. 3A), implying a positive role for [2Fe-2S]-IscR in the serum resistance of *K. pneumoniae*. To confirm this result further, we performed a complementation study. As shown in Fig. 3B, the introduction of pIscR, but not pACYC184 or pIscR_3CA_, into the AP001 strain increased the bacterial survival rate, to a similar level compared with that of WT [pACYC184], after serum treatment. These results supported the hypothesis that [2Fe-2S]-IscR activates the expression of CPS to increase *K. pneumoniae* resistance to normal human serum.

### IscR has a regulatory role in iron acquisition systems

**Figure 4:**
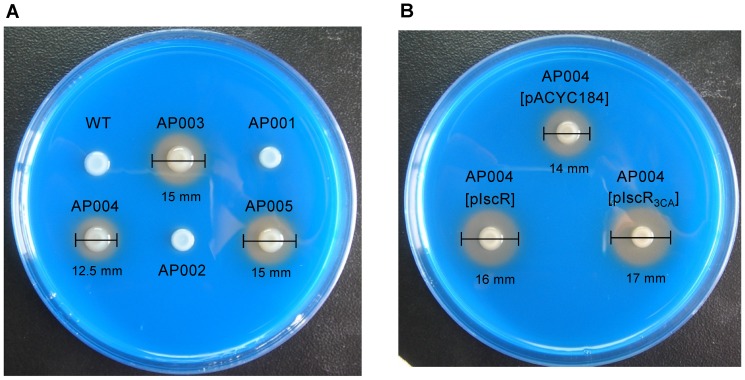
Deletion of *iscR* decreases *K. pneumoniae* Δ*fur* siderophore production assessed using CAS assay. All assayed bacterial mutants (A) and the complement strains (B) are indicated. The halos around the colonies correspond to the iron-chelating activity of siderophores in bacteria were measured after 24 h of incubation at 37°C. The assay was independently repeated at least five times, and the differences among strains are consistent.

In *E. coli*, both Fur and IscR play important roles in the maintenance of cellular iron homeostasis [12], [13]. To analyse whether IscR regulates iron acquisition in *K. pneumoniae*, we performed a chrome azurol S (CAS) assay to assess siderophore secretion in *K. pneumoniae* strains. As shown in Fig. 4A, no apparent siderophore secretion was detected in the WT, AP001, or AP002 strains. Moreover, as in our previous report [10], deletion of *fur* clearly increased halo formation on the CAS plate. However, the halo was reduced in the AP003 (Δ*fur*) strain background by the further deletion of *iscR*, indicating the positive role of IscR in iron acquisition. Furthermore, no obvious difference in siderophore secretion was found between the AP003 and AP005 (Δ*fur*- *iscR*_3CA_) strains, suggesting that the IscR regulation of iron acquisition does not require the [2Fe-2S] cluster.

To verify whether apo-IscR activates iron acquisition, we introduced pACYC184, pIscR, or pIscR_3CA_ into the AP004 (Δ*fur*-Δ*iscR*) strain and performed a CAS assay. As shown in Fig. 4B, the introduction of both pIscR and pIscR_3CA_ increased the halo phenotype on the CAS plate compared with that of the vector-only control. These results confirmed that IscR activates siderophore secretion in a [2Fe-2S] cluster-independent manner.

To further investigate the regulatory effect of IscR on iron acquisition, we used quantitative reverse transcription polymerase chain reaction (qRT-PCR) to measure the expression of genes corresponding to the eight putative iron acquisition systems in the indicated *K. pneumoniae* strains. As shown in Table 1, messenger RNA (mRNA) levels of genes (*fhuA*, *iucA*, and *sitA*) corresponding to three iron acquisition systems were increased more than 2-fold in the AP005 strain as compared with that in the AP004 strain. To further confirm this result, pACYC184, pIscR_3CA_, or pIscR were respectively introduced into the AP004 strain, to avoid the effects of Fur, and the transcription of *fhuA*, *iucA*, and *sitA* were measured. The introduction of pIscR_3CA_ into AP004 apparently increased the transcription of *fhuA*, *iucA*, and *sitA* compared with that in the AP004 strain carrying pACYC184 only (Table 1). These results implied that apo-IscR activates the transcription of *fhu*, *iuc*, and *sit* to increase iron acquisition in *K. pneumoniae*. Besides, the introduction of pIscR into AP004 also slightly increased transcription of *fhu*, *iuc*, and *sit* (Table 1).

**Table 1:** qRT-PCR analyses of the expression of iron-acquisition genes in *K. pneumoniae* strains.

Systems	Gene	RNA expression ratio b		
		AP005/AP004 a	AP004 [pIscR_3CA_]/AP004 [pACYC184]	AP004 [pIscR]/AP004 [pACYC184]
Fe^3+^				
Ferrichrome	*fhuA*	2.17 ± 0.31	2.83 ± 0.14	1.78 ± 0.42
Aerobactin	*iucA*	2.24 ± 0.12	3.23 ± 0.15	1.84 ± 0.22
Enterobactin	*fepA*	1.07 ± 0.38	ND c	ND
	*fepB*	1.11 ± 0.09	ND	ND
	*entC*	0.80 ± 0.09	ND	ND
Ferric citrate	*fecA*	1.23 ± 0.52	ND	ND
	*fecE*	1.74 ± 0.25	ND	ND
Salmochelin	*iroB*	1.92 ± 0.13	ND	ND
Heme	*hmuR*	1.58 ± 0.22	ND	ND
Fe^2+^				
Ferrous iron	*feoB*	1.35 ± 0.36	ND	ND
	*sitA*	3.16 ± 0.01	3.68 ± 0.11	2.27 ± 0.23

a AP004, CG43S3Δ*fur*-Δ*iscR*; AP005, CG43S3Δ*fur-iscR*_3CA_.

b Mean expression ratio (±SD) of AP005 relative to AP004, AP004 [pIscR_3CA_] relative to AP004 [pACYC184], or AP004 [pIscR] relative to AP004 [pACYC184].

c ND, not determined.

### IscR_3CA_ directly binds the promoter region of *fhuA*, *iucA*, and *sitA*

**Figure 5:**
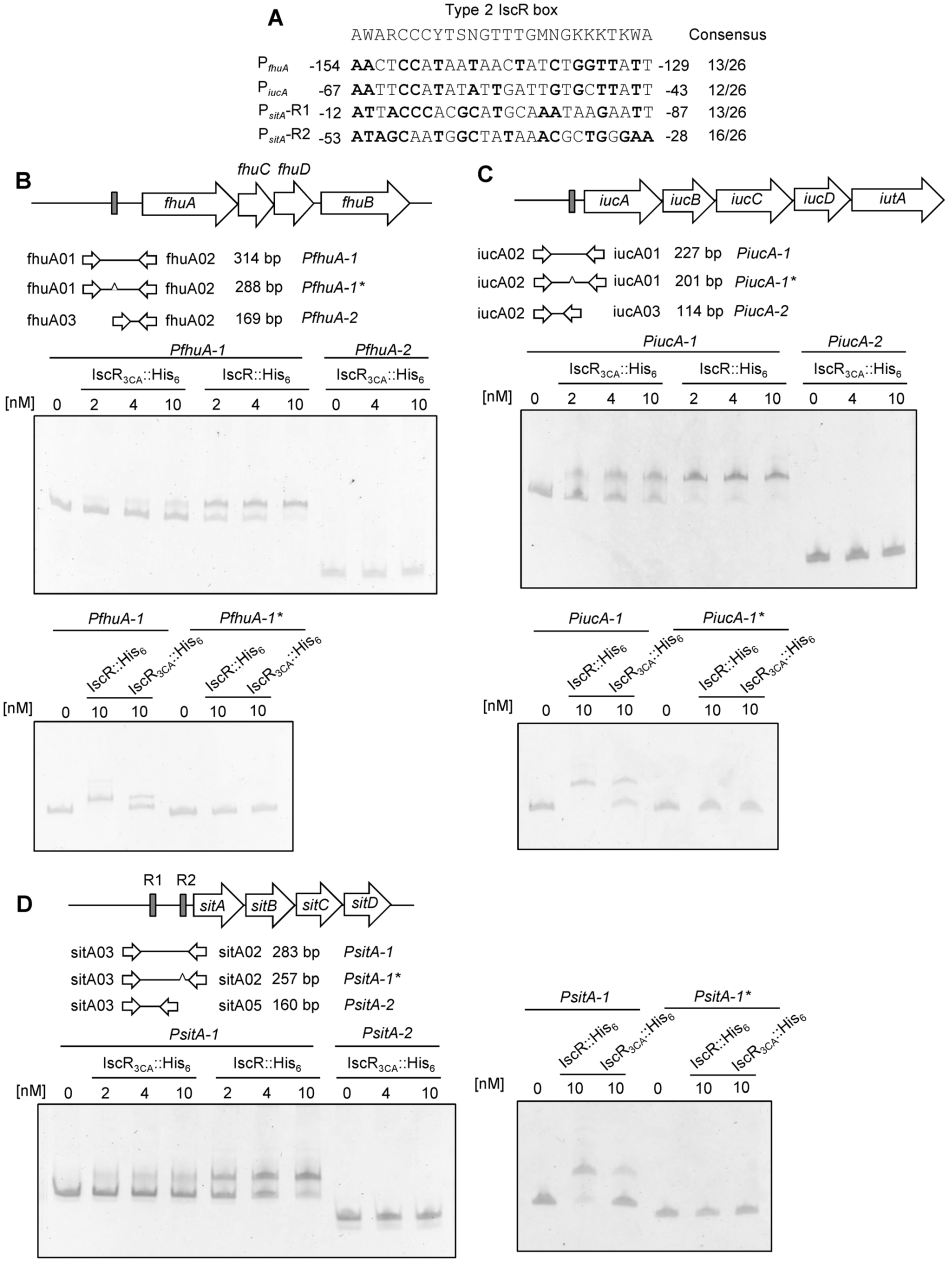
IscR_3CA_::His_6_ binds directly to P*_fhuA_*, P*_iucA_*, and P*_sitA_*. (A) DNA sequence alignment between the *E. coli* type 2 IscR box and the putative IscR binding sequence in the upstream regions of *fhuA*, *iucA*, and *sitA*. Positions identical to the consensus sequences are bolded. Diagrammatic representation of the *fhuA* (B), *iucA* (C), and *sitA* (D) loci. The large arrows represent the open reading frames. The primer sets used in PCR amplification of the DNA probes are indicated, and the numbers denote the DNA amplified length. The predicted IscR boxes is deleted and indicated by a caret. The grey boxes indicate the predicted type 2 IscR box. Different concentrations of purified IscR_3CA_::His_6_ were incubated with 5 ng of various DNA fragments of the upstream regions of indicated genes. Following incubation at room temperature for 30 min, the mixtures were analysed on a 5% non-denaturing polyacrylamide gel. The gel was stained with SYBR Green I dye and photographed.

Apo-IscR has been demonstrated to bind the type 2 IscR box in IscR-regulated promoter sequences directly in *E. coli* [19]. Analysis of the promoter regions of *fhuA*, *iucA*, and *sitA* revealed consensus sequences of the *E. coli* type 2 IscR box. As shown in Fig. 5A, the predicted type 2 IscR boxes are located at −154 to −130 relative to the translation start site of *fhuA* and −67 to −43 relative to the translation start site of *iucA*. The predicted type 2 IscR boxes in P*_fhuA_* and P*_iucA_* have 50% (13/26 bp) and 46% (12/26 bp) homology, respectively, with the consensus sequence. In addition, two putative type 2 IscR boxes (R1 and R2) located at −112 to −87 and at −53 to −28 relative to the translation start site of *sitA* were found in P*_sitA_*. The R1 and R2 sites contain 50% (13/26 bp) and 61.5% (16/26 bp) homology, respectively, with the consensus sequence.

**Figure 6:**
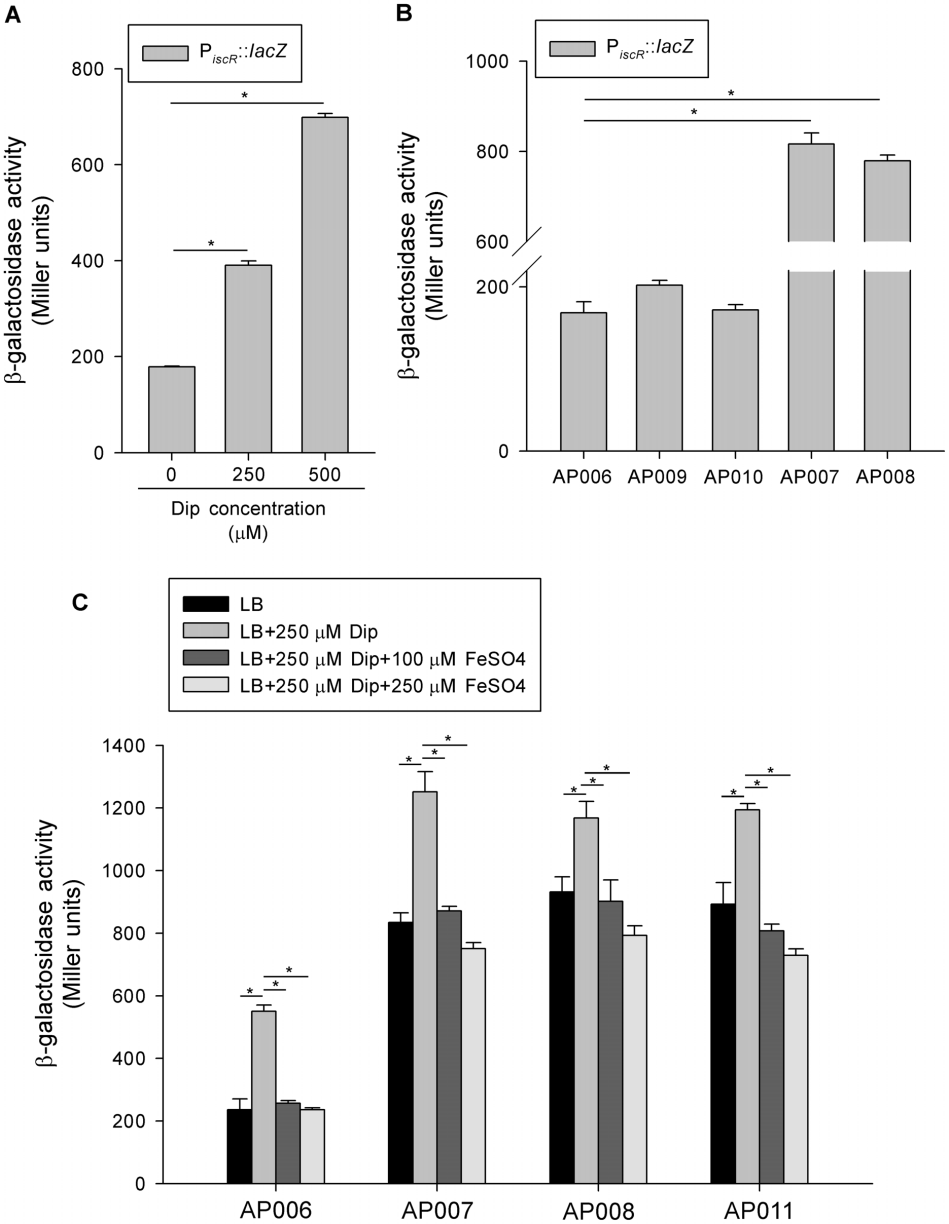
Regulation of *K. pneumoniae iscR* expression. (A) β-Galactosidase activity of *K. pneumoniae* AP006 (Δ*lacZ*) carrying the reporter plasmid piscRZ15 (P*_iscR_*::*lacZ*) were determined using log-phase cultures grown in the indicated concentrations of Dip. (B) The β-galactosidase activity of piscRZ15 was determined in the AP006 and isogenic strains, AP009 (Δ*lacZ-*Δ*fur*), AP010 (Δ*lacZ-*Δ*fur-*Δ*ryhB*), AP007 (Δ*lacZ-*Δ*iscR*), and AP008 (Δ*lacZ-iscR*_3CA_), using log-phase cultures grown in LB medium. (C) The β-galactosidase activity of piscRZ15 was determined in the AP006 and isogenic strains, AP007, AP008, and AP011 (Δ*lacZ-*Δ*fur-*Δ*iscR*), using log-phase cultures grown in the indicated media. Error bars indicate standard deviations. *P<0.01 compared with the indicated group.

To verify whether apo-IscR binds to these predicted type 2 IscR boxes, we performed an EMSA. As shown in Fig. 5B–D, both the purified IscR::His_6_ and IscR_3CA_::His_6_ were able to bind with the promoter regions of *fhuA*, *iucA*, and *sitA*, and IscR::His_6_ appeared to contain higher binding activities. Furthermore, IscR_3CA_::His_6_ did not bind *PfhuA-2* and *PiucA-2*, which lacked a region containing a putative IscR box (Fig. 6B–C). We also found that IscR_3CA_::His_6_ did not bind *PiucA-2*, which contained the R1 site but not the R2 site (Fig. 6D). To further confirm the importance of these predicted IscR boxes, recombinant IscR proteins were respectively interacted with these promoters lacking only the 26-bp predicted type 2 IscR box (PfhuA-1*, PiucA-1*, and PiucA-1*), and no obvious interaction was found. These results suggested that apo-IscR interacts directly with the promoters of *fhuA*, *iucA*, and *sitA* via the predicted type 2 IscR boxes.

### Regulatory control of *iscR* transcription in *K. pneumoniae*

To analyse whether environmental iron availability affects *K. pneumoniae iscR* expression, we grew AP006 in Luria-Bertani (LB) broth supplemented with increasing amounts of the iron chelator 2, 2-dipyridyl (Dip) and monitored the promoter activity of *iscR* using a LacZ reporter system [23]. As shown in Fig. 6A, the addition of 250 or 500 µM Dip to the growth medium increased *iscR* promoter (P*_iscR_*) activity by approximately 2-fold and 3.9-fold, respectively, indicating that the transcription of the *iscRSUA* operon was activated by iron limitation.

In *K. pneumoniae*, Fur and RyhB reportedly play crucial roles in gene regulation in response to cellular iron levels [10], [11]. Thus, we investigated whether Fur and RyhB regulate the activity of P*_iscR_*. As shown in Fig. 6B, the deletion of *fur* and the further deletion of *ryhB* in AP006 strain had no obvious effects on P*_iscR_* activity, whereas the deletion of *iscR* in AP006 strain activated P*_iscR_* activity by approximately 4.5-fold. In *E. coli,* IscR has been demonstrated to exert negative auto-regulation which requires the [2Fe-2S] cluster [20]. Thus, we measured P*_iscR_* activity in an AP006-derived strain, AP008 (Δ*lacZ-iscR*_3CA_), expressing a mutated IscR predicted to be defective in cluster binding [20]. As shown in Fig. 6B, the P*_iscR_* activity in AP008 was increased approximately 4.5-fold. This increase was comparable to that in AP007 (Δ*lacZ-*Δ*iscR*). Our results suggested that IscR inhibits the transcription of the *iscRSUA* operon in a [2Fe-2S] cluster-dependent manner in *K. pneumoniae*.

To investigate whether IscR is the sole regulator of *iscRSUA* transcription in response to iron availability, we monitored P*_iscR_* activity in the AP007 and AP008 strains in LB broth containing various levels of iron. As shown in Fig. 6C, the P*_iscR_* activity in AP006 was activated in LB broth supplemented with Dip, and the further addition of 100 or 250 µM FeSO_4_ reversed the activation. In the AP007 and AP008 strains, P*_iscR_* activity was increased compared with that of AP006. Nevertheless, the addition of Dip still activated P*_iscR_* activity, suggesting the presence of unknown factors, and the further addition of FeSO_4_ restored the effect.

To analyse whether Fur is responsible for this regulation, we measured P*_iscR_* activity in AP011 (Δ*lacZ*-Δ*fur*-Δ*iscR*) compared with that of AP007 at various iron levels and noted no obvious effect. These results indicated that in addition to IscR and Fur, other factors modulate the transcription of the *iscRSUA* operon in response to environmental iron availability.

## Discussion

Clinically isolated *K. pneumoniae* strains usually carry large amounts of CPS to resist engulfment by phagocytes and serum bactericidal factors [6], [24]. Therefore, tightly controlling CPS biosynthesis is critical for successful infection by *K. pneumoniae* [10], [11], [25]. We have previously shown that Fur represses the expression of mucoid factors RmpA and RcsA as well as the small RNA RyhB in response to environmental iron to decrease CPS biosynthesis indirectly in *K. pneumoniae* [10], [11]. In this study, we focused on IscR, a central regulator of iron metabolism, to analyse more thoroughly how external iron affects CPS biosynthesis. Our data indicated that IscR activates CPS biosynthesis in a Fe-S cluster-dependent manner (Fig. 1A and B). Moreover, IscR positively regulates the transcription of three transcription units in the *cps* gene cluster (Fig. 1C). Purified IscR::His_6_ also directly interacts with the promoter of *orf1–2*, possibly through the predicted type 1 IscR box (Fig. 2). These findings indicated that Fur and IscR exert negative and positive regulation, respectively, on CPS biosynthesis in response to external iron.

However, we hypothesize herein that Fur plays a major regulatory role because (i) *K. pneumoniae* grown under iron-replete conditions displayed decreased CPS levels, (ii) the AP004 strain produced elevated amounts of CPS compared with that of the WT and in the AP004 background, expression of IscR or IscR_3CA_ did not cause an obvious effect on CPS amount under iron-replete or iron-limited conditions (data not shown), and (iii) all three transcription units of the *cps* genes were obviously increased after *fur* deletion [10]. This result supported the notion that Fur plays a major regulatory role in the regulation of CPS biosynthesis. Nevertheless, the contribution of IscR could also be an important part of the iron network. On one hand, in iron-replete conditions, Fur indirectly represses the CPS production, however CPS is still needed for *K. pneumoniae* survival inside the host, so IscR may contribute in maintaining a higher basal expression level of the genes involved in CPS biosynthesis. On the other hand, in iron-limited conditions, the transcription of iron-acquisition genes is increased not only due to the de-repression of apo-Fur but also the activation of apo-IscR. Besides, in addition to iron level, oxidative stress has been demonstrated to signal Fur and IscR, and the reversible interconversion of Fe-S clusters makes them exquisite sensors of such stress [13], [18], [26]. Thus, the modulation of CPS levels by Fur and IscR under oxidative stress conditions could be predicted, but this hypothesis remains to be elucidated.

A BLAST search identified eight putative iron acquisition systems in the genome of *K. pneumoniae* CG43S3. Moreover, Fur directly represses the transcription of genes corresponding to six iron acquisition systems (*iucA*, *iroB*, *entC*, *hmuR*, *feoA*, and *fecA*) under iron-replete conditions [10]. In this study, we have shown that IscR directly activates the transcription of *fhuA*, *sitA*, and *iucA* (Table 1 and Fig. 5). Although *fur* deletion also led to increased expression of these three iron acquisition systems, an *in vivo* Fur titration assay demonstrated that Fur did not interact with the promoters of *fhuA* and *sitA* and also had a relatively lower binding affinity to *iucA* [10]. These findings indicated that Fur and IscR orchestrate the expression of iron acquisition systems in *K. pneumoniae*. In addition, Fur is suggested to play a major regulatory role because deletion of *fur*, but not *iscR* deletion, activated the secretion of siderophores, and the effect mediated by IscR was observed only in the *fur*-deleted background (Fig. 4).

Because IscR and Fur are both iron-responsive regulators which share strong overlap among downstream targets, we next investigated whether IscR and Fur are cross-regulated. In *E. coli*, the expression of the *iscR* is controlled not by Fur but by IscR itself [19], [20]. In *K. pneumoniae*, we found that P*_iscR_* activity is clearly activated by IscR but not influenced by Fur (Fig. 6), which is consistent to the finding in *E. coli*. Moreover, deletion of *iscR* did not affect the transcription of *fur* (data not shown), suggesting that in *K. pneumoniae*, IscR and Fur do not share cross-regulation. Furthermore, *E. coli* RyhB reportedly binds directly to the upstream region of *iscS* mRNA to decrease the stability of *iscRSUA* but not *iscR* mRNA, thereby leading to a stable secondary *iscR* structure and resulting in active translation [27]. Then, the increased IscR likely causes auto-repression of P*_iscR_* activity.

An analysis of the upstream region of *iscS* in *K. pneumoniae*, also identified a conserved sequence paired with RyhB (data not shown). However, the regulatory effect on P*_iscR_* activity mediated by RyhB was not obvious under our assay conditions (Fig. 6B). On the contrary, as shown in Fig. 6C, P*_iscR_* activity in AP007 and AP008 was still activated by iron depletion, which prompted us to verify whether IscR is the sole iron-responsive regulator that controls P*_iscR_* activity. However, in AP007, the addition of FeSO_4_ to iron-depleted medium still led to a reduction in P*_iscR_* activity. These results suggested that an unknown regulator, beside of IscR and Fur, represses the transcription of *iscR* in response to external iron. In addition to IscR, the [2Fe-2S] cluster is critical for regulation mediated by FNR and SoxR [27]. However, sequence analysis of the promoter region of *iscR* revealed no typical FNR and SoxR binding sites. On the contrary, we found a putative binding site of SoxS [28], an oxidative transcriptional regulator activated by SoxR, in P*_iscR_*. This putative SoxS binding site displays 79% (15/19 bp) homology with the consensus sequence and is located at position −144 to −126 relative to the translation start site of *iscR*. Therefore, we hypothesized that SoxS may be involved in the regulation of *iscR* in response to oxidative stress and will investigate this possibility in future studies.

IscR differs from other known Fe-S cluster-containing transcription factors such as FNR and SoxR because both apo- and holo-IscR regulate transcription and exhibit different DNA binding specificities [29]. Structural and biochemical studies have suggested that the ligation of the [2Fe-2S] cluster broadens the DNA binding specificity of IscR, thereby allowing holo-IscR to bind both type 1 and type 2 boxes, whereas apo-IscR binds only the type 2 box [29]. In the *K. pneumoniae* K2 *cps* gene cluster, we found a type 1 box in the promoter region of *galF*, and purified IscR::His_6_ could bind this motif *in vitro* (Fig. 2). In addition and as expected, the clusterless IscR_3CA_:His_6_, mimicking apo-IscR, showed no obvious binding affinity to P*_galF_*. On the contrary, all three promoters of the iron acquisition genes regulated by IscR contain predicted type 2 boxes, and both IscR::His_6_ and IscR_3CA_:His_6_ appeared to bind these boxes (Fig. 5). Although apo- and holo-IscR have been demonstrated to bind the type 2 box with similarly high affinity [21], EMSA revealed that IscR_3CA_:His_6_ displayed weaker binding (Fig. 5). In *E. coli*, the Fe-S cluster status of IscR is a key variable that regulates gene expression in response to iron availability [12]. Our results suggested that the transcription of *cps* and iron acquisition genes is regulated by not only the level of IscR but also the cellular ratio of apo- and holo-IscR.

Although IscR was first discovered as an auto-repressor of the *isc* operon, it is now known to be a global regulator that influences the expression of ∼40 genes in *E. coli* and ∼67 genes in *Vibrio vulnificus* [19], [30]. Although the regulon of IscR in *K. pneumoniae* has not been identified, our study provided evidence that IscR regulates CPS biosynthesis and iron acquisition, which are also regulated by Fur. Moreover, previous studies have shown that Fur controls type 3 fimbriae expression and biofilm formation in *K. pneumoniae* [31]. The possibility that IscR also participates in the regulation of these phenotypes is currently under investigation.

**Figure 7:**
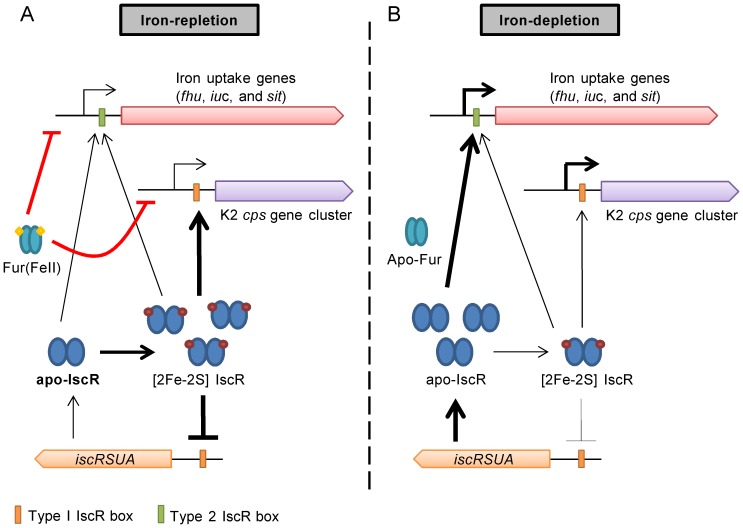
A proposed model for IscR regulation on the CPS biosynthesis and iron-acquisition genes in *K. pneumoniae*. (A) Under iron-replete conditions, and when the cellular supply of Fe-S clusters is sufficient, the holo-IscR exerts an auto-repression as well as activates the K2 *cps* genes and the iron-acquisition genes; however, Fur in complex with Fe(II) exerts a stronger repression on these genes (red lines). (B) Under iron-deplete conditions, the apo-IscR accumulates and preferably activates the transcription of the iron-acquisition genes but not the *cps* genes. Upon the de-repression of Fur, both the transcription of the iron-acquisition and *cps* genes are increased.

After infection, bacterial pathogens frequently encounter iron starvation and oxidative/nitric oxide stress conditions that are highly detrimental for maintaining Fe-S cluster homeostasis [32], [33], [34]. These conditions are predicted to influence the transcription of genes regulated by IscR. Thus, in response to these stress conditions, IscR may regulate CPS biosynthesis, iron acquisition systems, and other virulence factors in *K. pneumoniae* to facilitate bacterial persistence in the host. In this study, we demonstrated that external iron levels regulate CPS biosynthesis and iron acquisition systems through IscR in *K. pneumoniae* and proposed a working model (Fig. 7). In response to iron availability, IscR and Fur control the expression of downstream targets in a parallel and cooperative manner, which is predicted to play a crucial regulatory role during infection.

## Materials and Methods

### Bacterial strains, plasmids, and media

Bacterial strains and plasmids used in this study are listed in Table 2. Primers used in this study are list in Table 3. Bacteria were routinely cultured at 37°C in LB medium supplemented with appropriate antibiotics. The antibiotics used include ampicillin (100 µg/ml), kanamycin (25 µg/ml), streptomycin (500 µg/ml), and tetracycline (12.5 µg/ml).

**Table 2:** Bacterial strains and plasmids used in this study.

Strains or plasmids	Descriptions	Reference or source
*K. pneumoniae*		
CG43S3	CG43 Sm^r^	[41]
AP001	CG43S3Δ*iscR*	This study
AP002	CG43S3*iscR*_3CA_	This study
AP003	CG43S3Δ*fur*	[42]
AP004	CG43S3Δ*fur*-Δ*iscR*	This study
AP005	CG43S3Δ*fur-iscR*_3CA_	This study
AP006	CG43S3Δ*lacZ*	[23]
AP007	CG43S3Δ*lacZ-*Δ*iscR*	This study
AP008	CG43S3Δ*lacZ-iscR*_3CA_	This study
AP009	CG43S3Δ*lacZ-*Δ*fur*	This study
AP010	CG43S3Δ*lacZ*-Δ*fur*-Δ*ryhB*	This study
AP011	CG43S3Δ*lacZ*-Δ*fur*-Δ*iscR*	This study
AP012	CG43S3Δ*galU*	[35]
*E. coli*		
DH5α	*supE44*Δ*lacU169 (f80 lacZ*ΔM15)15)169*recA1 endA1 gyrA96 thi-1 relA1*	[43]
BL21(DE3)	*F^-^ ompT hsdS_B_[r_B_^−^m_B_^−^]gal dcm* [DE3]	New England Biolabs
S17-1 *λ pir*	*hsdR recA pro* RP4-2 [Tc::Mu; Km::Tn*7*] [*λpir*]	[37]
Plasmids		
pKAS46	Ap^r^ Km^r^, positive selection suicide vector, *rpsL*	[36]
yT&A	Ap^r^, TA cloning vector	Yeastern
pACYC184	Tc^r^Cm^r^, low copy number cloning vector	New England Biolabs
pIscR	Cm^r^, 980-bp fragment containing an *iscR* allele cloned into pACYC184	This study
pIscR_3CA_	Cm^r^, 980-bp fragment containing C92A, C98A and C104A mutant allele of *iscR* cloned into pACYC184	This study
placZ15	Cm^r^, promoter selection vector, *lacZ* ^+^	[23]
piscRZ15	Cm^r^, 312-bp fragment containing the region upstream of *iscR* cloned into placZ15	This study
pOrf12	Cm^r^, 500-bp fragment containing the region upstream of *Klebsiella K2 cps orf1-orf2 cloned into placZ15*	[23]
pOrf315	Cm^r^, 900-bp fragment containing the region upstream of *Klebsiella K2 cps orf3-orf15* cloned into placZ15	[23]
pOrf1617	Cm^r^, 300-bp fragment containing the region upstream of *Klebsiella K2 cps orf16-orf17* cloned into placZ15	[23]
pET30b-IscR	Km^r^, 654-bp fragment encoding full-length IscR cloned into pET30b	This study
pET30b-IscR_3CA_	Km^r^, 654-bp fragment encoding full-length C92A, C98A and C104A mutant allele of *iscR* cloned into pET30b	This study
piscR-pKAS46	Ap^r^Km^r^, 2.0 kb fragment containing *iscR* and its flanking regions cloned into pKAS46	This study
piscR_3CA_-pKAS46	Ap^r^Km^r^, 2.0 kb fragment containing full-length C92A, C98A and C104A mutant allele of *iscR* and its flanking regions cloned into pKAS46	This study

**Table 3:** Primers used in this study.

Primer	Sequence (5′→3′)	Enzyme cleaved
GT138	GGATCCTGCGTCGTCTGTTCACCC	*Bam*HI
GT139	AAGCTTCAATGCAAGGAATCAGGCA	*Hind*III
GT142	GGATCCCGGTCACGGCATAA	*Bam*HI
GT143	AGATCTAGTTGAACATCCTGCGCGGC	*Bgl*II
GT206	CTGAGCGCCGCCCTTGCCCTGGGCACG	
GT207	GGCGGCGATAAAGCCCTGACTCACG	
GT215	GCTCGAGGGCGCGCAGTTTAACGTCAAT	*Nhe*I
GT216	GCATATGAGACTGACATCTAAAGGGCG	*Xho*I
GT241	CGAGCTCCCATACCGGCAACATGGG	*Sac*I
GT242	CTCTAGACGCGCCATCTCGCTTTCC	*Xba*I
CC02	GGATCCTCTCATGTCTTACTTAACC	*Bam*HI
CC03	GGATCCAGGACGCGATTGAC	*Bam*HI
GT200	TCAGCGGCTCGTTCCTTTGC	
GT201	TCTCACTTCTGACTGTTGGTAAAAGGG	
YCC02	ACTGGATCCTGCGACCGGAATAACC	*Bam*HI
fhuA01	GAAGCTTGTCGCGGGCTGGATCAAG	*Hind*III
fhuA02	CGGATCCCGCAGCGAGTGATTTGGC	*Bam*HI
fhuA03	AATTCAGCCTTCGACCGGC	
fhuA04	AAAGAGCCGTTAACTTTTTTAC	
fhuA05	TAATTCAGCCTTCGACCGG	
iucA01	GGCAAAGTCATAGTCAAACAGATC	
iucA02	GAATCACCAGTACAGGGATCG	
iucA03	ACAACATCGTGACGTCTCTTGA	
iucA04	TTATAAAATAAATTTTATCAATCC	
sitA02	GGTGTAGCATAGGATCCCTC	*Bam*HI
sitA03	ATTTCTTGATCTTCGCCCGG	
sitA05	GTTATATAGCACAAGCTATTTAT	
sitA06	ATTAACCACACCATTGCGAG	
galF01	TCAGCGGCTCGTTCCTTTG	
cc02	GGATCCTCTCATGTCTTACTTAACC	*Bam*HI

For RT-qPCR	Sequence (5′→3′)	TaqMan probes	Target
RT11	GGTAGGGGAGCGTTCTGTAA	67	23S rRNA
RT12	TCAGCATTCGCACTTCTGAT		
GT56	ACCCCGCCAGCTTTAACTT	3	*entC*
GT57	TGTCCTTCTTTACGCAGCAG		
GT58	CAACCTGAACAGCGATTTCC	20	*fecA*
GT59	TCGGCGCTCTCTTTAACAGT		
GT62	CAGATGTCAGCGCAGATCC	20	*feoB*
GT63	CATAGGCCCGGCTGTAGA		
GT64	AAAGAGATTGGCCTCGAGTTT	20	*fepA*
GT59	TCGGCGCTCTCTTTAACAGT		
GT66	AATAAACAGCTCGTTTCGTTAAAAG	160	*fepB*
GT67	GTATAGACCAGGGCGGTCAC		
GT68	GTTTGGTCGTATCGCCTGAC	3	*fhuA*
GT69	GGAAGGTGAAGTCAGTTTTATCG		
GT72	TGATGACCTACCTGCAGTACCA	20	*hmuR*
GT73	GAGCCGAGGTTCCAGGAG		
GT74	CGGAGGAACATTCGTCAAA	84	*iroB*
GT75	TTCGGAATCTAAGCCTGGTG		
GT78	TCTCCCGGCTTATTGTTGATA	67	*iucA*
GT79	GGAAGGTTTCGCAACTGGT		
GT82	GAAGATCCGTCAGACGATGG	20	*sitA*
GT83	TAGTCGCGGGCCAGATAG		

### Construction of the deletion of *iscR* mutants

Specific gene deletion of *iscR* was introduced into *K. pneumoniae* CG43S3 using an allelic exchange strategy as previously described [35]. In brief, two approximately 1000 bp DNA fragments flanking both sides of *iscR* were cloned into the suicide vector pKAS46 [36], a suicide vector containing *rpsL*, which allows positive selection with streptomycin for vector loss. The resulting plasmid was then mobilized from *E. coli* S17-1λ*pir* [37] to *K. pneumoniae* CG43S3 or CG43S3-derived strains by conjugation. The transconjugants, with the plasmid integrated into the chromosome via homologous recombination, were selected with ampicillin and kanamycin on M9 agar plates. Several of the colonies were grown in LB broth supplemented with 500 µg/mL of streptomycin to log phase at 37°C and then spread onto an LB agar plate containing 500 µg/mL of streptomycin. The streptomycin-resistant and kanamycin-sensitive colonies were selected, and the deletion was verified by PCR and Southern hybridization (data not shown). The resulting *K. pneumoniae* mutants are listed in Table 2.

### Construction of the pIscR complementation plasmid and the pIscR_3CA_ mutant plasmid

To obtain the complementation plasmid (pIscR), a DNA fragment containing the promoter and coding sequence of *iscR* was amplified by PCR using the primer pair GT138/GT139 (Table 3) and cloned into the pACYC184 shuttle vector. The pIscR_3CA_ plasmid, which carried the mutant allele encoding IscR with the C92A, C98A, and C104A mutations, was constructed using the inverse-PCR method. Briefly, the pIscR plasmid was used as the PCR template to generate the mutant allele with the primer pair GT206/GT207 (Table 3). The recovered PCR product was treated with *Dpn*I for 2 h, subjected to T4 polynucleotide kinase treatment, and self-ligated with T4 DNA ligase. The ligation product was transformed into *E. coli* DH5α. The pIscR_3CA_ plasmid was subsequently confirmed by sequence analysis.

### Construction of a *K. pneumoniae iscR_3CA_* mutant

A DNA fragment carrying *iscR* and approximately 1000 bp adjacent regions on either side was amplified by PCR using primer pairs GT241/GT242 (Table 3) and cloned into yT&A. The resulting plasmid was used as the template for the inverse-PCR with the primer pair GT206/GT207 (Table 3) to generate a mutant *iscR* allele encoding the C92A, C98A and C104A mutations. Subsequently, the mutant allele of *iscR* was subcloned into pKAS46 and confirmed by DNA sequencing. Then, the plasmid was mobilized from *E. coli* S17-1 λ*pir* to the *K. pneumoniae* AP001 strain by conjugation, and the subsequent selection was performed as described above.

### Extraction and quantification of CPS

CPS was extracted and quantified as previously described [38]. The glucuronic acid content, represents the amount of *K. pneumoniae* K2 CPS, was determined from a standard curve of glucuronic acid (Sigma-Aldrich) and expressed as micrograms per 10^9^ c.f.u. [39].

### Measurement of promoter activity

The promoter-reporter plasmids, pOrf12, pOrf315, pOrf1617, and piscRZ15 were individually mobilized into *K. pneumoniae* strains by conjugation from *E. coli* S17-1 λ*pir*. The bacteria were grown to logarithmic phase in LB broth or indicated medium, and the β-galactosidase activity was measured as previously described [23].

### Bacterial survival in serum

Normal human serum, pooled from healthy volunteers, was divided into equal volumes and stored at −70°C before use. Bacterial survival in serum was determined as previously described [35]. First, the bacteria were grown to log phase in LB broth and the viable bacterial concentration was adjusted to 1×10^6^ c.f.u./ml. Next, 1 ml of the cultures was washed twice using phosphate-buffered saline (PBS) and resuspended in 1 ml PBS. A mixture containing 250 µl of the cell suspension and 750 µl of pooled human serum was incubated at 37°C for 15 min. The number of viable bacteria was then determined by plate counting. The survival rate was expressed as the number of viable bacteria treated with human serum compared with the number of viable bacteria pretreatment. The 0% survival of *K. pneumoniae* AP012 (Δ*galU*) served as a negative control.

### Purification of IscR::His_6_ and IscR_3CA_::His_6_

The coding regions of *iscR* and *iscR*_3CA_ were amplified using the primer pair GT215/GT216 (Table 3) and cloned into the *Nhe*I/*Xho*I site in pET30b (Novagen, 205 Madison, Wis). The resulting plasmids (pET30b-IscR and pET30b-IscR_3CA_, respectively) were then transformed into *E. coli* BL21(DE3)[pLysS] (Invitrogen, USA), and overproduction of the recombinant proteins IscR::His_6_ and IscR_3CA_::His_6_, respectively, were induced by the addition of 1 mM IPTG for 3 h at 37°C. The cell pellets were washed and resuspended in cold binding buffer (20 mM sodium phosphate, 0.5 M NaCl, 5 mM imidazole, pH 7.4). The cells were then broken by sonication and the cell pellets were removed by centrifugation at 14000 rpm for 10 min at 4°C. The recombinant proteins were then purified from the soluble fraction of the total cell lysate by affinity chromatography using His-Bind resin (Novagen, Madison, Wis) according to the manufacturer's instructions. The nonbinding proteins were washed away using binding buffer and the recombinant proteins were eluted by elution buffer (20 mM sodium phosphate, 0.5 M NaCl, 500 mM imidazole, pH 7.4). Finally, the purified proteins were dialyzed against TEGX buffer [20 mM Tris-HCl (pH 8.0), 0.5 mM EDTA (pH 8.0), 10% (v/v) glycerol, 0.2% Triton-X 100] containing 0.1 mM NaCl at 4°C overnight. Subsequently, the dialyzed protein was checked for purity by SDS-PAGE and stored for up to two weeks at 4°C. The purified protein was transparent and no obvious precipitation was observed after storage.

### EMSA

DNA fragments of the putative promoter regions *galF*, *fhuA*, *iucA*, and *sitA* were amplified with Pfu polymerase using specific primer sets (Table 3) to generate DNA probes for EMSA (*PgalF-1* and *PgalF-2* for *galF*; *PfhuA-1* and *PfhuA-2* for *fhuA*; *PiucA-1* and *PiucA-2* for *iucA*; and *PsitA-1* and *PsitA-2* for *sitA*). To obtain probes that lacked a putative IscR box, the DNA fragments were respectively amplified with Taq polymerase using the above-described primer sets (Table 3) and then cloned into yT&A to generate the plasmids pgalF, pfhuA, piucA, and psitA for subsequent mutagenesis via the inverse-PCR method using the primer pairs GT201/galF01, fhuA04/fhuA05, iucA03/iucA04, and sitA05/sitA06, respectively. The resulting mutant plasmids, pgalF*, pfhuA*, piucA*, and psitA*, respectively, were amplified with specific primer sets to generate the DNA fragments PgalF-1*, PfhuA-1*, PiucA-1*, and PsitA-1* for DNA probes in EMSA.

For the EMSA, the purified IscR::His_6_ and IscR_3CA_::His_6_ proteins were incubated with 5-ng DNA in a 10 µl solution containing 4 mM Tris-HCl (pH 7.4), 10 mM KCl, 100 mM dithiothreitol, and 10 µg/ml BSA at 37°C for 30 min. The samples were then loaded onto a native gel of 5% nondenaturing polyacrylamide in 0.5× TB buffer (45 mM Tris-HCl, pH 8.0, 45 mM boric acid). Gels were electrophoresed with a 20-mA current at 4°C and then stained with SYBR Green I dye (Invitrogen). The assay was repeated in at least 3 independent experiments.

### qRT-PCR

Total RNAs were isolated from early-exponential-phase grown bacteria cells by use of the RNeasy midi-column (QIAGEN) according to the manufacturer's instructions. RNA was DNase-treated with RNase-free DNase I (MoBioPlus) to eliminate DNA contamination. RNA of 100-ng was reverse-transcribed with the Transcriptor First Strand cDNA Synthesis Kit (Roche) using random primers. qRT-PCR was performed in a Roche LightCycler 1.5 Instrument using LightCycler TaqMan Master (Roche). Primers and probes were designed for selected target sequences using Universal ProbeLibrary Assay Design Center (Roche-applied science) and listed in Table 3. Data were analyzed using the real time PCR software of Roche LightCycler 1.5 Instrument. Relative gene expressions were quantified using the comparative threshold cycle 2^−ΔΔCT^ method with 23S rRNA as the endogenous reference.

### CAS assay

The CAS assay was performed according to the method described by Schwyn and Neilands [40]. Each of the bacterial strain was grown overnight in LB medium, and then 5 µl of culture was added onto a CAS agar plate. After 24 h incubation at 37°C, the effects of the bacterial siderophore production could be observed. Siderophore production was apparent as a halo around the colonies; the absence of a halo indicated the inability to produce siderophores.

### Statistical methods

An unpaired t-test was used to determine the statistical significance and values of *P*<0.01 were considered significant. The results of CPS quantification, β-galactosidase activity, serum survival rate, and qRT-PCR analysis were performed in triplicate and independently repeated at least three times, and the mean activity and standard deviation are presented.

### Ethics statement

For isolation of normal human serum from healthy volunteers, the procedure and the respective consent documents were approved by the Ethics Committee of the China Medical University Hospital, Taichung, Taiwan. All healthy volunteers provided written informed consent.

## Supporting Information

Figure S1 **Single-copy complementation of** ***iscR*** **but not** ***iscR*** **_3CA_ in the AP001 strain restores native production levels of CPS.** CPS levels of the *K. pneumoniae* strains, as indicated, grown in LB broth were determined as described in Materials and Methods (*P<0.01).
(TIF)

Methods S1 **Supporting Materials and Methods.**
(DOCX)
