# Supplementary figures and images for: IscR Regulation of Capsular Polysaccharide Biosynthesis and Iron-Acquisition Systems in Klebsiella pneumoniae CG43
Source: PLoS One. 2014 Sep 19;9(9):e107812. doi: 10.1371/journal.pone.0107812 (PMC4169559; doi:10.1371/journal.pone.0107812)

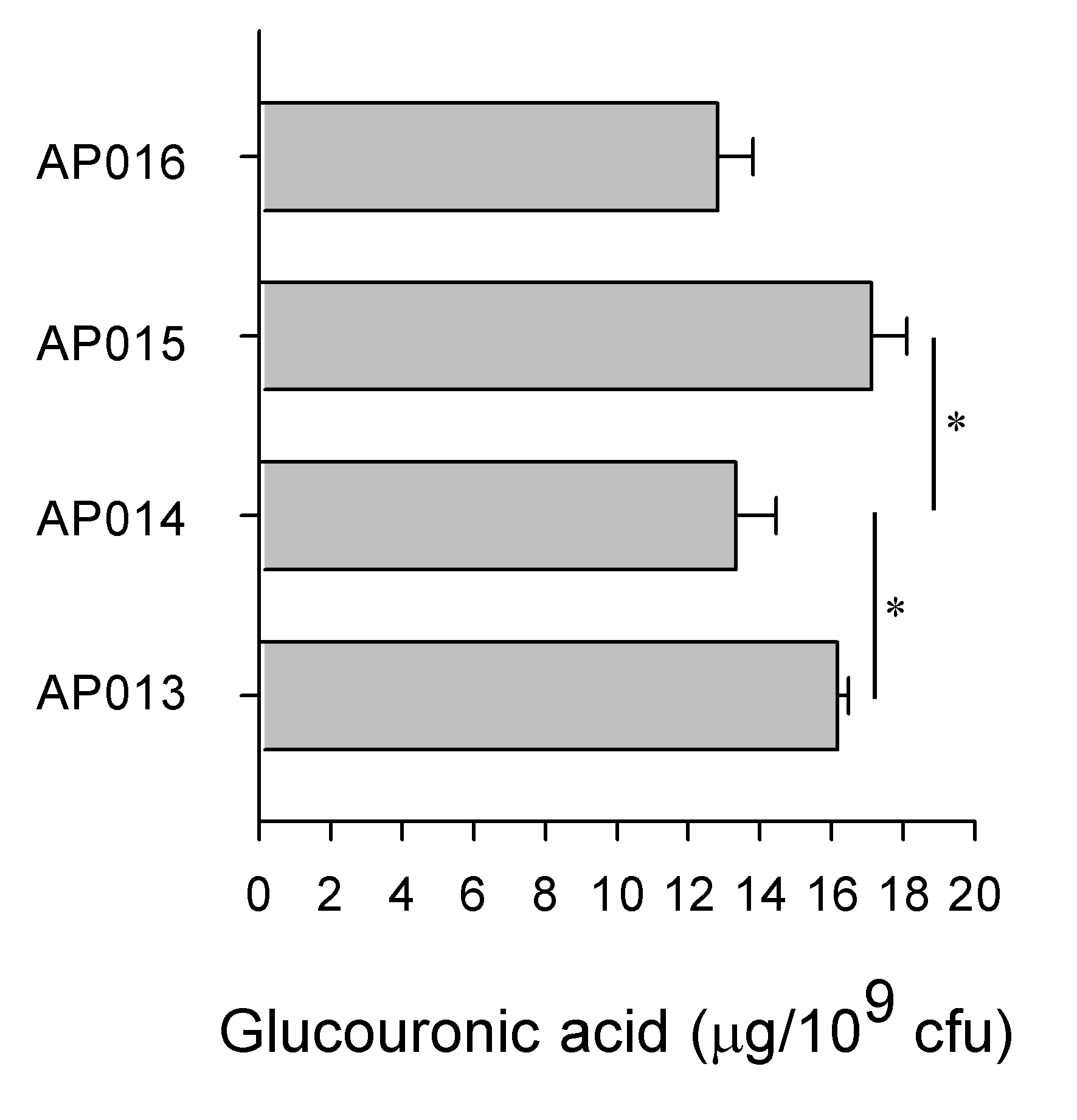

Supplement: Figure S1 — Single-copy complementation of iscR but not iscR 3CA in the AP001 strain restores native production levels of CPS. CPS levels of the K. pneumoniae strains, as indicated, grown in LB broth were determined as described in Materials and Methods (*P<0.01). (TIF) [file pone.0107812.s001.tif]
