# Supplementary material for: IscR Regulation of Capsular Polysaccharide Biosynthesis and Iron-Acquisition Systems in Klebsiella pneumoniae CG43
Source: PLoS One. 2014 Sep 19;9(9):e107812. doi: 10.1371/journal.pone.0107812 (PMC4169559; doi:10.1371/journal.pone.0107812)
Supplement: Methods S1 — Supporting Materials and Methods. (DOCX) [file pone.0107812.s002.docx]

**Supporting Information: Methods S1**

**Supporting Materials and Methods**

**Complementation *iscR* mutants in single copy integration**

To generate the single copy complemented strain of *iscR* mutants, 1.6-kb DNA fragments encompassing the promoter and coding sequence of *iscR* and *iscR_3CA_* or a 0.94-kb DNA fragment encompassing the promoter alone were amplified (using primers pairs GT241/GT139 for the 1.6-kb fragments and GT241/cc02 for the 0.94-kb fragment [Table 3]) and cloned into the suicide vector pKAS46 [36] to generate pKAS46_*iscR*com, pKAS46_*iscR_3CA_*com, and pKAS46_P*_iscR_*control respectively. The resulting plasmids were then mobilized from *E. coli* S17-1λ*pir* [37] to *K. pneumoniae* WT or AP001 strain by conjugation. The resulting transconjugants, with pKAS46_*iscR*com, pKAS46_*iscR_3CA_*com, and pKAS46_P*_iscR_*control integrated into the chromosome via homologous recombination, were selected with ampicillin and kanamycin on M9 agar plates. The complemented strains were designated AP015 (∆*iscR*_*iscR*com), AP016 (∆*iscR*_*iscR_3CA_*com), AP014 (∆*iscR*_control), and AP013 (WT_control) respectively.
